# Abstracts and Gleanings

**Published:** 1886-06-20

**Authors:** 


### ABSTRACTS AND GLEANINGS.

**American Medical Association.**—The following extracts, we clip from the report of the proceedings of the thirty-seventh annual meeting, held in St. Louis, Mo., May 4, 5, 6, and 7, 1886, as found in the *New York Medical Record* :

Tuesday, May 4.—Dr. William Brodie, of Detroit, Mich., delivered the president's address, in which, after referring to the facts that no epidemic disease had invaded the country during the last year; that a general state of health had been given to the members; then made special reference to the inroads made among our ranks by the removal by death of Drs. Bowling, John L. Atlee, and Austin Flint, of whom he gave brief biographical sketches. From this he passed to a sketch of the history of the organization of the Association, beginning with 1846, when it was conceived by Doctor Hayes, of Philadelphia ; special reference was made to the influence of the Association on the question of the elevation of the standard of medical education. The influence of the Code, as sustained by the Association, was dwelt upon, and the chief interest in this part of the address centered in the statement that the movement in the State of New York against it was due chiefly to financial considerations. He entered into a history of the International Congress, and closed with an expression of the belief that the general sentiment of the profession was in favor of adopting the report of the committee, and when adopted that personal feeling should yield to the common good of the profession.

Wednesday, May 5.—Dr. Nicholas Senn, of Milwaukee, Wis., delivered the address of the chairman of the Section of Surgery and Anatomy, in which he directed special attention to the present state of abdominal surgery by a condensed account of the advancement which had been made within the last few years in that department. He limited his remarks to the consideration of such injuries and lesions of the abdominal organs as were liable to be presented to physicians and general surgeons, aiming particularly to point out the limitations of abdominal operations, and to draw a distinct line between feasibility and justifiability. The first topic was penetrating wounds of the abdomen. The chairman mentioned the distinctions between punctured and gunshot wounds, and spoke of these as important with reference to diagnosis and treatment. Explorative laparotomy had for its objects positive diagnosis, arrest of hemorrhage, restoration of breach of continuity, and removal of extravasations. Dr. Senn prefers the bichloride to carbolic acid, and for uniting divided ends of intestine the Czerny-Lembert suture was the only one which should be used. If any doubt exists concerning the aseptic condition of the peritoneal cavity, a drainage tube should be inserted. He insisted upon the justifiableness of explorative laparotomy in all doubtful cases. Laparo-colotomy was the next topic, which was followed by that of subcutaneous laceration of intestines. Then came intestinal obstruction. The treatment of this condition by laparotomy was in its infancy, yet sufficient good results had been obtained to stimulate additional efforts in studying its limit of application. The results would improve as soon as physicians recognized the insufficiency of the expectant plan of treatment. Intussusception and enterostenosis followed, and for internal strangulation the rules given by J. Greig Smith were endorsed. These were followed by enterectomy, rupture of the diaphragm, and treatment of peritonitis by abdominal section and drainage. The last had been most successful in cases where the disease had not become diffused, and where the original cause could be removed. Tubercular peritonitis was considered next, and this was followed by gastrotomy, which was of questionable propriety in cases of malignant disease, but was recommended in all cases of non-malignant stricture. Pylorectomy, duodenostomy, and jejunostomy were the last topics considered. The general conclusion was that abdominal surgery was a legacy of inestimable value, left to us by McDowell, Gross, and Sims, that it should be cherished and studied, and that our knowledge of it should be carefully cultivated.

Dr. Henry F. Campbell, of Georgia, ex-president, was invited to take a seat upon the platform.

Dr. S. C. Gordon, of Portland, Me., then delivered the address of the chairman of the Section on Obstetrics and Diseases of Women, in which he said that nothing remarkable had been done or discovered in these departments during the last year. He could only emphasize matters which had been alluded to, and perhaps weigh inferences which had been made on topics that had been more or less discussed. With reference to attending cases of labor after having made an autopsy or while in attendance upon cases of puerperal fever, etc., he believed that the weight of opinion sustained the view that with proper regimen and absolute cleanliness the daily work of the physician could be safely continued without delay or anxiety. For the obstinate vomiting of pregnancy no one measure was of so much value as focible dilatation of the cervical canal below the internal os. In producing premature labor he recommended the use of the bougie, and when done on account of disproportionate head the eighth month was the most favorable time. In the department of gynecology only one theme was introduced, and that was the removal of uterine appendages included under the three operations known as Battey's, Tait's, and Hegar's. After making some remarks, the chairman spoke particularly of hysteria and its relation to diseases of the uterine appendages. This part of this subject was discussed at much length, and accompanied by the histories of illustrative cases in which cure had been effected by the operation. He had removed the uterine appendages in 25 cases, and with only one death. The conclusions reached by the speaker were based upon his study of the subject and the favorable results obtained in these cases.

Dr. A. L. Gihon, of Washington, chairman of the Rush Memorial Committee, reported that in obedience to the resolutions adopted by the Association at its last annual meeting, the committee had been instituted by the appointment of one member from each of the States, Territories, and National services represented in the Association, and that the standing committee thus organized would proceed at once to collect funds for the erection of a statue of Dr. Benjamin Rush, in the city of Washington, by the members of the profession of medicine in the United States. The secretary of the committee is George H. Rohe, of Baltimore, and the treasurer, J. M. Toner, of Washington, D. C.

Thursday, May 6.—The secretary read the report of the committee on nominations, signed by P. O. Hooper, of Arkansas, chairman, and William Wile, of Connecticut, secretary. President—E. H. Gregory, of St. Louis, Mo. Vice-presidents—B. H. Miller, Stillwater, Minn.; W. B. Welch, Arkansas; William H. Pancoast, Philadelphia; William C. Wile, Connecticut.

The next place of meeting, Chicago, Ill., and the time the first Tuesday in June, 1887. Chairman committee of arrangements, Charles G. Smith.

Dr. A. L. Gihon read the report of the committee on the president's address, signed by John H. Murphy, of St. Paul, chairman, and A. Garcelon, of Maine. First, in their opinion, it was proper and desirable that this Association should without delay memorialize Congress in behalf of appointing a scientific commission consisting of three to visit the habitats of yellow fever and determine the value and the claims of Drs. Carmona and Freire, who maintain that they have discovered means of preventing attacks of the disease. * * * That the Association should emphatically denounce the endorsement, by certificate, or advertisement, or testimonial, or by any form whatsoever, of proprietary remedies and appliances, and it should instruct the judicial council to take action in all such cases without the formal presentation of charges, and that it should be regarded as a stigma to endorse the use of such proprietary means for the relief and cure of disease. Seventh, that it is desirable that the Association should appoint a committee at this meeting, to consider the advisability of changing the organic law, by establishing branches or by some other plan, and to report at the next annual meeting. Eighth, they earnestly re-echo the wish of the president that the members of the profession will cordially co-operate to make the next International Medical Congress attractive and instructive to the foreign members, sacrificing personal piques and disappointments to contribute to that success which has been unconditionally pledged in the invitation extended to the Congress to meet in the United States in 1887.

Dr. J. T. Whittaker, of Cincinnati, then delivered the address of the chairman of the Section on Practice of Medicine. There are three planes in the history of medicine: First, the study of the symptoms or appearance of disease; second, the observation of the effects or lesions of disease; third, investigation into the cause of disease. The etiology of acute infections is comprized under the single term, bacteriology, for it has now been demonstrated that pathogenic micro-organisms do exist beyond dispute in distinct and definite entity. The speaker then considered the theory of spontaneous generation, the claims of convertibility of all germs, the question of mutability or immutability, the deviations that do occur, the doctrine that the germ, not the form, is essential, the morphology of bacteria, the destruction of bacteria, the formation of zooglea, the subject of pure culture soils, the necessities with reference to the temperature and the need of oxygen, the fecundity of micro-organisms, the topic of spores and saprophytes, the mode of invasion and dissemination, the effects upon the tissues, their presence in neoplasms, in giant cells, in the blood. The question of immunity was studied, and also the manner in which micro-organisms, produce the phenomena of disease. The address concluded with a brief reference to the subject of ptomaines.

Friday, May 7.—The Secretary read a preamble and resolutions presented by Dr. J. McF. Gaston, setting forth the claims of inoculation as a preventive of yellow fever and providing for the appointment of a committee to memoralize Congress in favor of passing the pending bill for a scientific commission to investigate this subject by proceeding to South America and Mexico. Adopted unanimously.

Complimentary and appropriate remarks were made by President elect Gregory, and by President Brodie, and the Association adjourned to meet in Chicago on the first Tuesday in June, 1887.

**Saving Condemned Limbs.**—Dr. Sampson Gamgee, in the *British Medical Journal*, says: A surgeon's responsibilities are never more delicate and weighty than when he is called upon to review a decision to amputate a limb. To the cases elsewhere published, in which, from inability to coincide with proposed amputations, I have successfully adopted measures to save limbs, brief notes of a more recent case may be added preliminary to comment.

In compliance with an urgent call into the country, I visited a patient whose leg was to be amputated next day. I gathered from the medical attendant that a fracture through the right ankle had occurred about a fortnight previously; great swelling having supervened, a considerable number of leeches were applied, and then padded wooden splints. When these were removed, after four or five days' intense suffering, a large wound was exposed on the inner aspect of the limb, which was much swollen. Splints were then discontinued, carbolic acid lotion applied, and amputation advised after consultation with a hospital surgeon.

I found the limb from foot to knee greatly swoollen and tense, of deep red color, and exquisitely sensitive to the slightest touch. The skin, to the extent of several square inches over the tendo Achillis and on the outer part of the leg, threatened disorganization; and on the inner side of the ankle was an irregularly shaped, deeply excavated wound, measuring from side to side five inches and five-eighths, from above downwards three inches and seven-eighths, and seemingly penetrating in its depressed centre into the ankle-joint. The patient's constitutional state was sound, but pain and want of sleep had produced considerable exhaustion. Unable to concur in the proposed amputation, I advised twenty-four hours' delay, to test the effect of the treatment which I proceeded to carry out. Carefully supporting the limb, I raised the foot and placed on the outer side from the lower third of the thigh downwards a double millboard splint, gummed, and padded three inches thick with dry absorbent gauze and cotton tissue. The splint embraced the sole of the foot, which, as well as the whole inner side of the limb, was padded with equally thick and perfectly smooth folds of the tissue. The whole was now smoothly bandaged with long spirals and without reverses, and with equable but decided pressure. Four six-yard bandages were used. The limb was then placed on the outer side in a swing, and I visited the patient with the medical attendant the next day. Pain had been much less, and the loosened bandages denoted great subsidence in the swelling. Taking the limb out of the swing, the bandages on the inner aspect were cut, and the absorbent tissue, soaked with a great quantity of matter, removed; but the millboard splint on the outer side was not touched for some days. The skin was paler and much less sensitive and tense. The same dressing was repeated on the following three days. The improvement locally and constitutionally was then so marked that the question of amputation was not reopened; and henceforward the dressing was only renewed every other day. A considerable slough separated from over the tendo Achillis and the outer part of the leg, and the absorbent tissue before application was lightly sprinkled with some of the following lotion:

| | | |
|---|---|---|
| ℞. | Sulphate zinc.................................. | grs. iv, |
| | Methylated spts. wine......................... | ℥ ss, |
| | Glycerin............. ...................... | ℥ j, |
| | Water........................... ......... | ℥ j. |

The wound surface was occasionlly touched with solid sulphate of copper. The dense deposit in the leg above the wound softened, and gave evidence of subcutaneous pus, which was carried off by drainage-tubes, one of which was passed several inches up the inner side of the leg, by corkscrew movement, from beneath the upper edge of the wound. Another tube was introduced through a small incision just outside the middle of the tibia. The outer ends of the drainage-tubes were made to project out of the dressings into a tin tray containing dry earth, which could be changed whenever necessary. The more perfectly to immobilize the foot and ankle, a bracket millboard splint was secured on the outer side, so as to embrace the leg just below the calf, and also the outer edge of the foot. Later on gummed millboard splints were placed on the inner and posterior aspects; and it may give some idea of the amount of padding to state that at each dressing half a pound of the absorbent gauze and cotton tissue was used. For the first six weeks the dressing was renewed every other day, after that on Mondays and Thursdays. The patient left her bed for the couch at the end of two months; in another fortnight she removed down stairs, and then the wound on the inner side of the ankle was solidly healed, with fair and steadily improving movement in the joint.

The recovery of the limb was due to a variety of agencies, chief amongst which were immobility, position, pressure, infrequent and dry dressings, with drainage by absorbent tissue, glycerin and tubes. It was remarkable how the very tender limb, which at first could scarcely bear the slightest touch, became comfortable under equable elastic pressure, physiological position, and absolute rest. I have elsewhere recorded a case in which a patient with a tender swollen leg, similarly treated, spontaneously expressed himself in these words: "It is wonderful how I can bear the limb handled now, and I could not stand a feather touching it last night." Glycerin in such cases acts as an antiputrescent, and by its great affinity for water powerfully aids absorbent tissues in securing perfect surface drainage, and keeping parts clean and sweet without the use of water. The plan of wound treatment, based on physiological principles, which I have enjoyed the frequent privilege of illustrating in these columns, was strikingly exemplified in the case above related. The limb was in such a condition that it was impossible to foretell its recovery with certainty, and it was only by the utmost care at every dressing that the result was attained. But I was from the first hopeful. In a large proportion of cases in hospital and private practice in which I have been called upon to amputate, I have not touched the knife and have spared the limb. A clear conception of physiological principles, unprejudiced application and combination of therapeutic resources according to the circumstances of particular cases, gentle, yet firm painstaking, yet not meddlesome manipulation, are very powerful agencies in saving condemned limbs.—*Medical and Surgical Reporter.*

**Tubercular Phthisis; Treatment of First Stage.**—Nutritious food, a well-ventilated room and a certain amount of outdoor exercise must be insisted upon from the beginning; tonic measures are also indicated. Cod-liver oil, as a rule, is not well borne when there is a high range of temperature. The time for that useful agent will come later. The cough owing to its tendency to induce hemoptysis, should be held in check. The popular "hydrocyanic mixture" used in the public hospitals of New York will be useful for this purpose. The formula is about this:

℞. Morphiæ sulphat..........................gr. 1-16,
Acid. hydrocyan. dil........................gtt. j,
Syr. tolutan.................................ʒ j.

M. One dose. To be repeated four to six times daily.

Cyanide of potassium (gr. 1-16) may be substituted for the hydrocyanic acid. Morphia, in small doses, is not apt to be attended with evil results, but if it be objectionable, a few drops of chlorodyne or an occasional drop or two of wine of antimony will serve to keep the cough in check. Later along, when the expectoration becomes profuse, we must give stimulating expectorants to enable the patient to get rid of the excessive secretion. If an attack of hemoptysis should supervene, the patient should at once be placed in the recumbent position and kept perfectly quiet, and should swallow small pieces of cracked ice every few minutes, or an ice-bag may be placed over the chest, care being taken not to allow freezing of the surface to occur. I have found these measures, together with the use of this formula, invariably successful in the treatment of hemoptysis:

℞. Ext. ergot. fl..............................fl ʒ vj,

Tinct. opii deodorat..........................fl ʒ ij.

M. Dose 20 to 25 drops, to be repeated every fifteen minutes until hemoptysis ceases.

For the nightsweats the patient should take every night at bed-time one-sixtieth of a grain of atropine in solution or one-thirtieth to one-twentieth of a grain of picrotoxine.

Aromatic sulphuric acid and oxide of zinc are also useful for colliquative sweats. There is no little difference of opinion in regard to the value of local measures in the treatment of phthisis. Of late there seems to be a tendency to discard them altogether among many practitioners. Pain is very seldom a prominent symptom in phthisis. If it should be severe, moderate counter-irritation, cautiously applied, may be found to promote the comfort of the patient. Vienna paste (equal parts of potassa and lime made into a paste with alcohol) is strongly recommended by no less an authority than M. Jaccound. Whether or not such applications promote the resolution of diseased lung tissue or tend to promote phthisical processes is quite problematical. It strikes me that a tri-weekly painting of the affected parts with tincture of iodine or a vigorous friction with the liniment of ammonia before retiring, together with careful protection of the surface by means of warm flannels, will accomplish about as much as we can expect from topical measures.

Very little has been determined positively with regard to the value of different resorts for consumptive patients, and such treatment is as yet largely experimental. Physicians in the Eastern States usually advise their patients to go to the pine regions of Southern Georgia or to Florida at the beginning of winter, returning North again gradually as spring approaches. I would advise to exchange the changeable climate and humid atmosphere of the Atlantic seaboard for the high, bracing latitude of Colorado.—*James K. Crook, M.D., in Weekly Medical Review.*

**Sulphate of Sodium.**—Especially in our country, whenever a neutral salt seems indicated as a purgative, Epsom salt—sulphate of magnesia—is usually the remedy employed, while sulphate of sodium—Glauber salt—is neglected. One reason for this may be found in the fact that Glauber salt is very cheap, and that it is used mainly in horses. Not long ago we advised a patient to take Glauber salt, but when the patient asked his apothecary for it, the latter ridiculed the idea, and said that this salt was used for horses only.

Epsom salt cannot be administered to young children, because its action on the delicate mucous membrane of the alimentary canal of the young is too harsh; but Glauber salt can be given with immunity. This fact alone should convince everybody that in most cases sulphate of sodium is preferable to sulphate of magnesia.

Nothnagel, a year or two ago, investigated the action of neutral salts on the intestines. He found that sulphate of sodium—Glauber salt—was the mildest of all; that it first caused a very slight redness, and later induced depletion by emptying the congested capillaries and producing a slightly watery stool. In the inflammatory intestinal disorders of childhood, as for instance in cholera infantum, if administered early in moderate doses, it acts as a specific, while sulphate of magnesia induces a severe congestion of the intestinal mucous membrane, and generally destroys the epithelial lining. Experience has proved this observation to be correct, for, generally, whenever, especially a larger dose of Epsom salt has been given, the patient will suffer from constipation, or at least from sluggishness of the bowels, for several days afterwards.

The action of Glauber salt is so certain and at the same time so much a true laxative—not causing congestion and not injuring the epithelial lining—that a much smaller dose is generally needed than in the case of the magnesia sulphate. In most cases from one-half to a teaspoonful, if administered to an adult, largely diluted with water—as all neutral salts should be if the intention is purgation—one hour before breakfast, will induce one or two motions of the bowels about half an hour after the meal, and this result will be brought about without the least griping and without any after-effect, as constipation, so often observed as a consequence of the magnesia salt.

We publish these remarks mainly for the benefit of those who imagine, because Glauber salt is used by veterinary surgeons, that its use would not be indicated in the case of human beings, for it is used for horses only on account of its cheapness. In every case it should be preferred to Epsom salt, for the reasons given.—*Med. and Surgical Reporter.*

**Belladonna as a Remedy in Acute and Chronic Catarrh.**—Dr. E. F. Allen, of Hartford, Conn., kindly sends us the following notes: The prevalence of catarrh in this climate, and the unsatisfactory results following the usual methods of treatment, prompted me, a few years ago, to experiment with other and less commonly used remedies. Basing my theory on the known physological action of belladonna, I have used it extensively in cases of acute and chronic catarrah of the naso-pharangeal mucous membrane, and in my hands it has yielded more satisfactory results than all other remedies. In a climate where "hard colds" are so frequently experienced, and so often lead to graver diseases, it appears to me as necessary to seek for satisfactory remedies in this as in typhoid or the major diseases.

I can positively affirm that in belladonna we have a drug more effectual in aborting or curing acute coryza or catarrh, than any remedy it has been my good fortune to hear of. Taking the disease in the begining, or before the discharge has reached the purulent stage, this remedy is capable of effecting a cure—"tuto cito et jucunde."

I find minute doses more satisfactory than larger ones, Dr. Bartholow to the contrary, notwithstanding, and my practice is to give only about 2 drops in the twenty-four hours, just enough to keep a *slight* feeling of dryness in the throat: 2 drops in an ordinary glass of water, and let the patient take tea-spoonful doses every half hour, until a slight effect is produced, after which a teaspoonful every hour or so during the entire day, and (if possible) the night. Twenty-four hours usually suffice to so control the disease as to allow the patient to resume his ordinary occupations.—*The American Medical Digest*

**Hyoscamine in Choera.**—The efficacy of hyoscamine as a remedy in the treatment of chorea has been illustrated in a case under the care of Prof. DaCosta, at the Pennsylvania Hospital, U.S.A., and which is described in the Boston *Medical and Surgical Journal* for January 23d last. A boy, eleven years of age, was admitted into the hospital referred to, suffering with chorea to a degree that rendered him completely helpless; he was unable to walk or to feed himself, and he had not sufficient control over his powers of speech to convey even the nature of his wants. At night, also, complete muscular repose was not obtained, and his condition generally was a pitiable one. Hyoscamine was administered, at first in doses of one two-hundredth of a grain, this amount being after a short time doubled; three doses per day were given. The effect was most marked, the patient being able to leave his bed and walk about the ward in four days; and his condition underwent steady improvement, until, three weeks from the date of his admission, his muscular system had regained its normal condition, and locomotion was in every way natural. In a clinical lecture on this case Dr DaCosta explained that he was induced to resort to trial of hyoscamine in connection with it owing to the fact that the drug had been employed with such signal benefit in cases of tremor; and it is extremely satisfactory to find that the results obtained were of so encouraging a character. As compared with the method of treatment by arsenic, the time gained through employment of hyoscamine is very marked, but it remains to be seen how far the latter rather than the former remedy secures permanence of the effects produced so much earlier, and with such apparent ease.—*Medical Press and Circular.*

**Insomnia in the Aged.**—Dr. C. L. Dana (N. Y. P. G. M. S., *Bulletin* of the Clin. Soc. of) has found the information contained in the text books upon insomnia in the aged to be very slight in amount. Insomnia was not frequent in the aged, but when it was present it was sometimes very intractable. In his experience, iron did not relieve the anæmia of the aged so as to produce sleep. Alcohol with the food was another remedy, and many recommended hot gruel or hot milk with alcohol before going to bed. While alcohol would relieve some cases, there were others in which insomnia was increased. The bromides and chloral, even when given in enormous doses, often failed to give relief. Opium was another remedy. Good results had been obtained with a combination of cannabis indica and codeia; from five to six mimims of the fluid extract of cannabis indica with one-sixth to one-eighth of a grain of codeia might be used. One-fourth of a grain of the extract of cannabis indica taken alone was sometimes effective. As a rule, however, the combination with codeia, was preferable. Hyoscyamine was sometimes useful, but in nervous, flighty persons it would sometimes produce an actual delirium. Under ordinary circumstances the dose should not be increased above one-fortieth of a grain to obtain the desired effect. The effect of these remedies, he thought, had been increased by the addition of from two to three drops of tincture of aconite two or three times a day to relieve the tension of the blood-vessels. Tincture of valerian and compound spirits of lavendar sometimes acted like a charm in relieving insomnia. Large doses (℈j) of lupulin were also often effective.—*Epitome.*

**Bromine in Diphtheria.**—Dr. P. Hesse, of Griefswald, calls attention to the fact that bromine, as a local medicament in diphtheria, has held its own, for some twenty years, better than any other remedy, and recommends for inhalation the following solution:

℞. Pure bromine, } aa................1 to 2 parts,

Potassium bromide, }

Distilled water............................400 parts.

Without regarding bromine as a specific, Hesse maintains that, properly applied, it is less fallible than other medicaments. Certain precautions and limitations in the application of the treatment are indicated, viz :

1. Treatment to be instituted as early as possible.
2. Direct application by the brush should not be made.
3. The frequency of application, and the concentration of the solution used for inhalation should be conditioned by the severity of the case, and the degree of congestion induced in the pharyngeal mucous membrane.
4. The solution should always be fresh, and, the treatment should be accompanied by ice-cold applications to the neck.
5. The method is contra indicated in laryngeal diphtheria, in bronchitis, and in affections of the lungs.—*Deutsches Archiv. f. klin Med.* March 24, 1866.

**Vaccinization.**—Dr. Samuel W. Abbott, of Boston, (*Boston Medical and Surgical Journal*) in his report on the recent progress in public medicine refers to Dr. Titeca and Dr. Warlomont, of France, who believe that vaccination should be raised to the state or condition of an axiom ; in other words, that it may be made perfectly protective.

To be perfectly protective Dr. Titeca says that it should be practiced under the following conditions:

1. The employment of vaccine virus which has lost none of its vitality.
2. The number of inoculations should be in accordance with the individual vaccinal receptivity, even to the extent of the exhaustion of that condition.

The first of these conditions is fulfilled by the use of living (*vivant*) vaccine, collected at the time of the operation; the second by the operation to which Dr. Warlomont gives the name of vaccinization.

Every vaccination made in default of these conditions is deceptive, and to such faulty methods the anti-vaccination school owes its existence. Its faults and grievances will cease when the school of vaccination is recognized.

The author recommends the following practice:

1. Vaccinization of infants within six months of birth.

2. Revaccinization every ten years, when no epidemic is prevailing, and general revaccinization in time of epidemic.—*Epitome.*

**Convenience and Economy in the Use of Cocaine.**—The fact that cocaine solutions, unless freshly prepared, lose their anæsthetic efficacy, has led to much waste of this expensive product, inasmuch as it was not always practicable for the physician and surgeon to prepare extemporaneously a solution which should be sufficient only for the particular case in hand. Facilities for properly and economically applying the cocaine solution are not always at hand when needed, and much unnecessary annoyance and expense has been caused those practitioners using the drug in their practice.

Appliances, therefore, which tend to facilitate the application of cocaine and economize the quantity used, must be commended and meet with very general appreciation.

Such an appliance exists in the complete cocaine case before us, which is made by Messrs. Parke, Davis & Co. This case, which is handsomely gotten up in morocco, lined with velvet, contains five capsules each holding 1 gr. cocaine muriate in crystals, a vial, to contain a solution of cocaine, a minim pipette, a camel's-hair pencil, a hypodermic syringe and card containing formula and directions for making the two per cent. and four per cent. solutions muriate cocaine.

One great advantage of this case is that it enables physicians to prepare a *fresh* solution of cocaine, whenever desired, at a *minimum* expense. We believe that its convenience and utility will be readily recognized by the profession, and must congratulate these enterprising manufacturers on meeting so promptly the requirements of the medical profession.—*Indiana Medical Journal.*

**The Pasteur Mission.**—The Commission of Inquiry into the system carried out by M. Pasteur to prevent the development of hydrophobia, which has been appointed by the British Government, has now commenced its investigation. The commission consists of Sir H. Roscoe, M. P., who moved for the appointment of the Commission in the House of Commons, Sir James Paget, F.R.S., Dr. Burden Sanderson, F.R.S., Dr. Quain, F.R.S., Principal Veterinary Surgeon G. Fleming, of the Army Veterinary Department, Dr. Lauder Brunton, and Mr. Victor Horsely, F. R. C. S., of the Brown Institute, the latter acting as Secretary to the Commission. Sir H. Roscoe, Drs. Brunton and Sanderson, and Mr. Horsley are now in Paris, and before long the public may look forward to the issue of a report which will enable them to appreciate the value which properly attaches to the processes adopted by M. Pasteur. The composition of the Commission renders it quite unnecessary to indicate the nature of the evidence which should be sought for; but we may say that, so far, no reports which have come under our notice have given sufficient evidence that their compilers have eliminated the many sources of error which have to be dealt with before any trustworthy opinion can be expressed as to the methods carried out in Paris.—*Lancet*, May 1, 1886.

**Mag. Sulph. in Rheumatism.**—Dr. S. B. Chase, Osage, Iowa, writes in Iowa *Medical Reporter*: "Permit me to call the attention of my medical brethren to the familiar remedy at the head of this article, and ask them to give it a trial in muscular rheumatism. From personal experience I have much confidence in its remedial power in this vexatious complaint. Like the waters of the Jordan it is simple and not costly; and though not especially palatable is unusually well borne by the stomach, while the disease is more unendurable than was Naaman's.

"For two years its presence was an abiding infliction, resisting every remedy sought. On the causation theory of subalkaline blood, I commenced the use of mag. sulph. in broken doses, 5 to 10 gras. three times a day in cold water, immediately after eating, with almost magic results. In a few weeks I could take a full inspiration without pain, a thing I had not done for months; was able to dress myself unaided and walk without a cane—the power of my tormentor broken and every mindful vestige gone."

**Foreign Body in the Nose.**—Dr. John N. Mackenzie relates a case of foreign body in the nasal cavity of a child, the removal of which was facilitated by the use of cocaine. Dr. Mackenzie regretted to be obliged to intrude upon the Society's time by bringing before them the much talked over cocaine, but in this case it proved itself of so much service that he considered himself justified in calling attention to its value in such cases. When he saw the child there was a bit of bone very tightly wedged between the corrugated mucous membrane. An attempt to remove it with forceps failed. He applied cocaine to the tissue in order that its power of causing contraction might lessen the pressure and enabled him thus to remove the foreign particle. This effect was produced in a few minutes, and the bit of bone was easily removed.

Dr. H. Clinton McSherry had been in the habit of using cocain with most happy results in obstinate cases of epistaxis.—*Medical and Surgical Reporter*.

**Drying up the Milk.**—Dr. R. J. Russel (in Bell Co. Med. Society—*Texas Courier-Record*) says: In sixteen cases in which I have found it necessary to dry up the milk, or rather to prevent any secretion of milk, I have used nothing but fluid ext. ergot administered in drachm doses three times a day and have not failed, in a single instance, out of the whole number, to meet with perfect success; nor have I in any case, seen any ill effect from long continued use of large doses of ergot.

**On the Treatment of Ringworm.**—Dr. R. W. Leftwich writes to the Lancet: Last August a lady asked me to examine her nurse-maid's head. I did so, and found a well-marked patch of ring-worm about an inch and a half in diameter. The mistress was naturally unwilling to expose to the contagion her children, who presented no sign of the disorder, and almost equally unwilling to part with the girl for a time. After some reflection, I told her I thought the difficulty might be gotten over with only very slight risk to the children, and treated the case in the following way: Having cut the hair close to the scalp, all round the patch, I first painted it with an alcoholic solution of iodide of mercury—an old-fashion but excellent remedy, obtained by adding calomel to tincture of iodine, and using the supernatant colorless fluid. As soon as the slight soreness it produced had passed off I applied an iodine plaster, obtained from a formula in Beasley's book and attributed to Roderberg, an ounce of the plaster containing a half a drachm of solid iodine. This, spread on kid, was carefully applied to the patch, which it overlapped all round. At the end of a fortnight it was removed, and the ringworm appeared practically cured. To make sure, however, it was again painted with the above-mentioned solution, and a fresh plaster applied for another fortnight. Upon being taken off, the whole surface of the patch was found covered with short hairs. No other patch has made its appearance upon the head or elsewhere, and not one of the three children with whom the patient was in daily and hourly contact took the complaint. Possibly the plaster alone would have been sufficient, but I thought it safer to use the paint in addition, and I feared that if I used a more powerful plaster the irritation might tempt the patient to remove it. I might also have used a plaster containing oleate of murcury, but doubted whether it could be made sufficiently adhesive. The advantages of this mode of treatment are obvious enough, for by its means the risk of the disease being spread by actual contact, by means of caps and by the common use of hair-brushes, is reduced to minimum. I find no allusion to this method in the ordinary works on the subject, and therefore infer that, if not new, it is not widely known.—*American Practitioner and News.*

**Endometritis.**—Before the Glasgow Obstetrical and Gynecological Society, February 10th, Dr. Robert Bell read a paper on "Endometritis," in which he expounded some rather peculiar views regarding its etiology and pathological relations. He endeavored to make out that constipation of the bowels is a very common and important factor in its causation, especially in young women, leading not only to endometritis, but to displacements of the uterus and subsequent dysmenorrhea; and, moreover, is the most frequent cause of anæmia, "from the constant absorption of fetid matter in the blood, which destroys the health and even the vitality of the red corpuscles, thus reducing their number and quality." In regard to treatment, Doctor Bell advocated the ordinary means for the removal of uterine congestion found in the text-books, such as the prolonged hot-water douche at bedtime, and the use of his medicated tampons of glycerine, alum, and boracic acid, and the weekly introduction of iodine and carbolic acid into the interior of the uterus. Where constipation existed, "systematic and prolonged use of the enema."—*London Medical Press.*

**Remedies for Skin-Diseases in the Form of Spray.**—Dr. Hardaway (Journal of Cutaneous and Veneral Diseases, vol. iii., No. 4) has in generalized pruritus had good results from spraying a lotion of the following sort: Carbolic acid (three to four drachms), glycerin 1 ounce, and water, a pint. After the bottle of the atomiser has been filled, he sometimes directs the patient to add from 5 to 10 drops of oil of peppermint. The atomizer bottle should be thoroughly shaken before the bulb is compressed, in oder to diffuse the peppermint through the mixture, as otherwise it would merely float on the top. In many instances the spray is far superior to mopping or less troublesome, getting the remedy more evenly and uniformly applied over the surface, and usually giving more speedy relief.—*London Medical Record.*

**The Treatment of Varicocele.**—Dr. Robert F. Weir, Surg. to New York Hospital (*Medical Record*, March 20, 1886), gives the following conclusions based upon his own experience:

1. That for small varicoceles, there is nothing better than the single (or double) subcutaneous catgut ligature.
2. That for medium-sized varicoceles or for cases declining a more heroic operation, excision in careful hands is to be advised.
3. That for large varicoceles, for relapsed cases, and for those not very large, but with a much elongated scrotum, ablation of the scrotum with ligation of the veins is preferable.

**Treating Dysentery with Cocaine.**—We have received many letters inquiring about Dr. Winter's method of treating dysentery with cocaine. His treatment of a young child was as follows: Ten drops of an eight-per-cent. solution, in one drachm of water, were injected into the rectum, which relieved the tenesmus for two hours; then twenty drops of the same solution, in a drachm of water, were injected. This was repeated every five or six, or eight hours, according to indications.—*Medical Record.*

**Menthol in the Treatment of Urticaria and Pruritus.**—Among the myriad of remedies for these troublesome affections we have used this remedy for urticaria in three cases. Not only is the itching relieved for the time, but a cure seems to be effected. In pruritus ani, and in eczema, moistening the parts with menthol solution causes an immediate cessation of the pain. The solution should contain from two to ten grains of menthol to the ounce of water.—*Exchange.*

**Parthenine in the Treatment of Facial Neuralgia.**—Tovar has experimented with this alkaloid in cases of facial neuralgia (*Gazz. med. Ital.-Lombard.*) Giving a tenth of a grain every hour for four hours, and then decreasing the size and frequency of the dose, he cured rather a severe case in a week. Parthenine is obtained from *Parthenium hysterophorus*, an herb growing in Jamaica, where it is much used for cutaneous affections.—*New York Medical Journal.*

